# Items

**Published:** 1881-05

**Authors:** 


### Items.

THE ILLINOIS STATE MEDICAL SOCIETY.—The thirty-first annual meeting of the Illinois State Medical Society will be held in Chicago, in the hall of the Methodist Church Block, beginning at 10 a. m., on Tuesday, May 17.

The officers for the year are:

President, Geo. Wheeler Jones, M.D., Danville; First Vice-President, Wm. Hill, M.D., Bloomington; Second Vice-President, G. W. Nesbitt, M.D., Sycamore; Treasurer, J. H. Hollister, M.D., Chicago; Permanent Secretary, S. J. Jones, M.D., Chicago; Assistant Secretary, W. T. Montgomery, M.D., Chicago.

Committee of Arrangements: Dr. J. N. Hyde, Chairman, Chicago; Drs. N. Bridge, R. Park, E. F. Ingals, P. S. Hayes, Chicago.

The Standing Committees that are expected to report are—

Committee on Practical Medicine: E. P. Cook, Mendota; W. West, Belleville; G. W. Nesbitt, Sycamore.

Committee on Surgery: Chas. T. Parkes, Chicago; Wm. A. Byrd, Quincy; D. S. Booth, Sparta.

Committee on Obstetrics: H. Webster Jones, Chicago; W. S. Caldwell, Freeport; C. T. Reber, Shelbyville.

Committee on Gynæcology: J. W. Dora, Mattoon; Ellen A. Ingersoll, Canton; B. F. Crummer, Warren.

Committee on Ophthalmology and Otology: F. C. Hotz, Chicago; J. P. Johnson, Peoria.

Committee on Drugs and Medicines: Frank H. Davis, Chicago; W. C. Pipino, Quincy; W. G. Cochran, Farmer City.

Committee on Necrology: T. F. Worrell, Bloomington; G. W. Albin, Neoga; E. Ingals, Chicago.

Committee on State Register: E. P. Cook, Mendota; F. L. Matthews, Springfield; D. S. Booth, Sparta.

The Special Committees appointed to report are—

Dermatology and Syphilis: W. J. Maynard, Chicago.

Laryngeal Tumors: E. F. Ingals, Chicago.

Abnormal Thermal Conditions in Diseases and the Means of Controlling them: J. H. Hollister, Chicago.

Results in Fractures: C. C. Hunt, Dixon.

Volunteer papers will be presented by—

1. Permanent Removal of Hair by Electrolysis: By Plym. S. Hayes, M.D., of Chicago.

2. On the Present Status of Specialism in Medicine: By A. Reeves Jackson, M.D., of Chicago.

3. On the Surgical Anatomy of the Sheaths of the Tendons in the Palm of the Hand: By Roswell Park, M.D., of Chicago.

4. On Ringworm of the Scalp: By Wm. J. Maynard, M.D., of Chicago.

CHICAGO MEDICAL COLLEGE (MEDICAL DEPARTMENT OF THE NORTHWESTERN UNIVERSITY).—The annual commencement exercises of this institution were held on Tuesday, March 29, 1881, in Central Music Hall, Prof. Davis, Dean of the Faculty, presiding. The candidates for graduation occupied the front seats of the auditorium; next were the undergraduates of the medical classes, with many members of the classes in the College of Law and the College of Liberal Arts, while the rest of the large hall was crowded with citizens. On the platform, the Dean was accompanied by his colleagues of the medical faculty; Oliver Marcy, LL.D., Acting President of the University, most of the professors in the Colleges of Law and of Liberal Arts, and members of the Executive Committee of the Board of Trustees.

The exercises were opened with prayer by Prof. F. W. Fisk, of Chicago. Prof. W. E. Quine, Secretary of the Faculty, then read his report, which stated that the twenty-second annual session of the college had been characterized by a degree of success in every department, which had been gratifying to the faculty and creditable to the students. During the session of twenty-six weeks, or six full months, clinical instruction had been given daily to full classes in the Mercy Hospital and College, and to small classes for individual training in the dispensary, and practical lessons in anatomy, chemistry, and microscopy in the several laboratories, which, with the didactic lectures and demonstrations in the several branches, make an aggregate of 2,688 lessons and lectures of one hour each. The amount and variety of instruction thus given, both practical and scientific, and its methodical arrangement, were claimed to be equal, if not superior, to that given in any other medical college in this country. The supply of anatomical material and other means of illustration had been ample, and the clinical resources more than could be utilized. Forty-one advanced students had received special certificates for satisfactory service in surgical dressing, and twenty-seven students received certificates for service as clinical assistants in the dispensary. The whole number of persons entered upon the college register and receiving instruction during the college year was 191. At the commencement of the present college year, as in each of the preceding years since the autumn of 1876, several of those who applied for admission were refused from inability to comply with the requirements in regard to general education. In the regular examinations of classes, twenty per cent. of the freshmen, or first year students, failed to reach the junior or second year class; and twenty per cent. of the juniors failed to qualify fully for entering the senior class. Some of these will make up their deficiencies during the summer vacation and retain their class standing, but about thirty per cent. of the whole number fail to obtain the desired diploma. According to this report, the medical faculty of the university aims to pay strict attention to the *proficiency* of its graduates rather than to their number. The Dean, Prof. N. S. Davis, then conferred the degree of Doctor of Medicine upon those members of the senior class who had been recommended by the faculty for that purpose, accompanying the delivery of the diplomas by a brief and impressive charge concerning the nature of the duties and responsibilities they were about to assume.

E. W. Andrews, of the graduating class, responded in a feeling manner, alluding to the close association that existed between professor and student in the pursuit of medical knowledge, and paying a regretful farewell to the dean and faculty in behalf of the class.

The following is the list of graduates:

E. W. Andrews, J. H. Bacon, R. T. Bailey, A. E. Baldwin, J. H. Bates, E. D. Bergeron, Frank Billings, A. T. Blackburn, G. C. Blish, A. H. Burr, C. E. Cook, W. G. Cook, D. C. Davies, W. L. Dayton, J. O. Everett, M. L. Felkner, J. A. Fordyce, W. S. Gates, D. A. Hadley, H. B. Hemenway, B. U. Jacob, F. S. Johnson, J. L. Williamson, E. A. Kegley, A. T. King, W. J. Latta, P. A. Letourneau, Richard Melms, F. T. Nye, A. E. Okey, J. L. Priestman, M. Z. Putnam, E. B. Righter, George Rivard, H. O. Rockwell, J. F. Rood, Fernando Roys, J. D. Simpson, F. A. Snyder, Heman Spaulding, J. H. Stowell, D. H. Sullivan, De W. Townsend, O. M. Vaughan, M. H. West.

Dr. H. A. Kelso received an honorary degree.

Dr. E. C. Helm, Dr. J. J. Larkin, and Dr. L. T. Potter, received special diplomas for one year each of post graduate service in the hospital.

Prizes were conferred for the best thesis, on J. L. Williamson, of Milwaukee, and H. B. Hemenway. The anatomical prizes were received by W. Porter and G. A. Wells, Prof. Rea making the presentation with a humorous and poetical speech. The Dr. Edwards prize was taken by W. E. Morgan, and that of Dr. Senn, of Milwaukee, by A. M. Vail, of Kansas. J. H. Bates also received a prize offered by Prof. Jenks for the best essay on a gynæcological subject. The chemistry prizes were awarded to W. A. Mann, M. D. Burbank and G. B. Eaton.

The valedictory address was delivered by Dr. Hollister, who spoke of the inherent feebleness of man, but he was possessed of perception, reason and judgment, which were perpetual food for thought. The experience of 6,000 years told of the mountains that were in the path of knowledge. He spoke of the achievements of medical science in the last century as being greater than ever before in the world's history. A medical knowledge could not be measured to-day by that of twenty-years ago; it now demands preliminary education and great culture. The Chicago Medical College had been organized to test the graded system, and its success proved the efficiency of the new plan. Seven of its professors were its own graduates, and the institution was out of debt. He then spoke of the fact that other colleges were adopting the graded system, and read letters from China and Japan, which said the American system had been adopted in distant lands with official sanction. Applications for admission here had been received from Russia and Australia. He congratulated the graduating class, for the Faculty, and trusted they would take up their life-work, and always acquit themselves like men.

The exercises were interspersed with music, and the young men of the graduating class received many beautiful floral tributes.

The fifteenth annual reunion and banquet of the Alumni Association of the College was held Tuesday evening, March 29, at the Tremont house. The graduates, including the class whose graduating exercises were held during the afternoon, gathered to the number of about 150 in the club-rooms, where a preliminary reunion and business meeting was held. An address of welcome was delivered by Dr. R. Park, in which he stated that a departure had been made from previous years, as the banquet was this year tendered by the Faculty to the Alumni.

Dr. F. E. Waxham read the necrological report, and the President, Dr. W. E. Quine, read his annual address. The election of officers resulted, on the report of a committee on nominations, as follows: President, D. A. K. Steele, '73; Vice-Presidents, Frank Kimball, '80, and Frank Billings, '81; Secretary and Treasurer, C. W. Earle, '70; Necrologist, F. E. Waxham, '78. Letters were read from absentees, and a social reunion followed. At about 10 o'clock the old and young doctors adjourned to the dining hall, where an excellent dinner was served. After the dinner had been discussed at length the speaking began, the prescribed toasts being as follows: "Welcome," responded to by Prof. N. S. Davis; "The Old Boy," Dr. J. E. Link, of '62; "Reminiscences of a 10-year-old Graduate," C. W. Earle, of '70; "The Badger as a Practitioner," Clark Gapen, of '75; "The Last Cathartic," J. A.

Fordyce, of '81. The responses were full of medical witticisms, and were followed by impromptu toasts, responses, songs and anecdotes, which occupied the time until an early hour in the morning.

THE TREATMENT OF PYROSIS OR WATER BRASH.—M. Ory gives, in *La France Médicale*, the following formulæ, which may be usefully prescribed to complete the rational treatment by proper regimen, milk and vegetable diet, and alkaline drinks.

Jeannel advises a tablespoonful, from two to four times a day, of the following mixture:

℞. Rhei pulv ............................ ʒ iiss

Sodii bicarbonat............................gr. xxx

Syrupi.......................................f℥ iiss

Aquæ menthæ viridis.......................f℥ viiss. M.

Each tablespoonful represents about seven and a half grains of rhubarb.

In the Pharmaceutical Formulary of London is the following mixture of chalk:

℞. Cretæ præparatæ,
Sacchari albi...........................each ℥ ss
Acaciæ pulv....................................ʒ ij. M.

Or the compound chalk powder—

℞. Cretæ præparatæ................................ʒ iij

Canellæ pulv....................................ʒ ij.

Tormentill. radicis pulv.,

Acaciæ pulv ............................each ʒ iss

Piperis longi pulv...............................gr. xv. M.

Dose, fifteen to thirty grains.

This powder, according to Bouchut, is also of service in cases of chronic diarrhœa.

The following powder has also been prescribed with success:

℞. Magnesiæ,
Sacchari pulv...........................each ʒ ij
Bismuthi subnitrat...............................gr xv
Sodii bicarbonat.................................gr.xxv. M.

Divid. in chart. x.

Take one powder at the beginning of each of the two principal meals.

Franck has advised the following:

℞. Magnesii carbonat..........................gr.xxx
Rhei pulv.,
Canellæ pulv..............................each gr.viiss. M.
Take in two doses.

M. Ory also recalls the composition of Warner's Cordial Liquor:

℞. Rhei pulv.................................ʒ iss

Sennæ pulv..................................ʒ j

Croci pulv..................................gr xv

Glycyrrhiz. pulv............................ʒ j

Uvar. passar................................℥ iij

Alcoholis (21°).............................f.℥ xij. M.

Digest for fifteen days and filter.

Administer a teaspoonful morning and evening, a short time before each meal.

M. Bouchardat suggests the following in the treatment of this affection:

℞. Rhei pulv.,
Magnes. calcinat. pulv.....................each gr.xxv
Opii pulv...................................gr.ss.
Divid. in chart. v.
Dose, one each day, before the principal meal.

Statistics of Medical Journals.—On the authority of Mr. Durean, one of the librarians of the Académie de Médecine, the present number of medical periodicals for France and its colonies is one hundred and forty seven, ninety-five of these being published in Paris, and fifty-two in the departments. The German empire publishes one hundred and thirty-three journals, Great Britain and Ireland sixty-nine, Austria fifty-four. Italy fifty-one, Belgium twenty-eight, Spain twenty-six, Russia twenty-six, Holland sixteen, Switzerland ten, Sweden and Norway nine, Denmark five, Portugal four, the Danish Principalities four, Turkey two, Greece one; the total for Europe being five hundred and eighty-three. In America there are one hundred and eighty-three journals, in Asia fifteen, in Oceanica four; the total for the various continents being seven hundred and eighty-five. The number of medical journals created since 1679 exceeds two thousand, five hundred.—*Lyon Médical.*

WONDERFUL MATERNAL IMPRESSION, IF TRUE.—Dr. Frank M. Ramsey, of Knoxville, Tennessee, writes to the Nashville *Journal of Medicine and Surgery*, April, 1881:

"Maternal impression once admitted, as I think it should be if records are properly made, I believe the fact will be established that at any period during gestation a mother may be impressed so as to leave an evidence on the embryo or fœtus.

"A negro woman, belonging to my own household, was in the seventh, perhaps eighth month of gestation, when she was thrown from a horse and fell in soft mud, except one leg in its lower third, across a hay-rod, or sapling used to bind hay on a wagon. Twenty-four hours afterward she aborted. The child had a well-defined ecchymosis on its leg, corresponding with the leg of the mother which struck the hay-rod, and the leg of the child was broken, though no mark, even, was made on the leg of the mother. This occurring under my own observation is sufficient to cause me to disbelieve that any particular time of gestation is more favorable to cause responsive effect on the embryo and fœtus than another."

AMERICAN GRADUATES IN MEDICINE IN 1881.—Jefferson Medical College (Phila.), 205; University of the City of New York 191; Rush Medical College (Chicago), 172; University of Nashville and Vanderbilt University, 168; College of Physicians and Surgeons (Baltimore), 154; Bellevue Hospital Medical College (N. Y.), 117; University of Pennsylvania, 110; Medical College of Ohio (Cincinnati), 103; University of Louisville, 100; Indiana Medical College (Indianapolis), 85; University of Maryland, 73; Louisville Medical College, 54; Nashville Medical College, 53; University of Buffalo, 48; Chicago Medical College, 45; Miami Medical College (Cincinnati), 34; Cincinnati College of Medicine and Surgery, 30; Michigan College of Medicine (Detroit), 28; Hospital College of Medicine (Louisville), 24; Woman's Medical College (Chicago), 17.

